# Fabrication and Characterization of Single Phase α-Alumina Membranes with Tunable Pore Diameters

**DOI:** 10.3390/ma8031350

**Published:** 2015-03-20

**Authors:** Tatsuya Masuda, Hidetaka Asoh, Satoshi Haraguchi, Sachiko Ono

**Affiliations:** 1 Graduate School of Engineering, Kogakuin University, 2665-1 Nakano, Hachioji, Tokyo 192-0015, Japan; E-Mails: bd12002@ns.kogakuin.ac.jp (T.M.); asoh@cc.kogakuin.ac.jp (H.A.); 2 Toshiba Corporation Power and Industrial Systems Research and Development Center, 1 Toshiba, Fuchu, Tokyo 183-8511, Japan; E-Mail: satoshi.haraguchi@toshiba.co.jp

**Keywords:** anodizing, porous anodic alumina film, membrane, crystallization, α-Al_2_O_3_, filtration property

## Abstract

Nanoporous and single phase α-alumina membranes with pore diameters tunable over a wide range of approximately 60–350 nm were successfully fabricated by optimizing the conditions for anodizing, subsequent detachment, and heat treatment. The pore diameter increased and the cell diameter shrunk upon crystallization to α-alumina by approximately 20% and 3%, respectively, in accordance with the 23% volume shrinkage resulting from the change in density associated with the transformation from the amorphous state to α-alumina. Nevertheless, flat α-alumina membranes, each with a diameter of 25 mm and a thickness of 50 μm, were obtained without thermal deformation. The α-alumina membranes exhibited high chemical resistance in various concentrated acidic and alkaline solutions as well as when exposed to high temperature steam under pressure. The Young’s modulus and hardness of the single phase α-alumina membranes formed by heat treatment at 1250 °C were notably decreased compared to the corresponding amorphous membranes, presumably because of the nodular crystallite structure of the cell walls and the substantial increase in porosity. Furthermore, when used for filtration, the α-alumina membrane exhibited a level of flux higher than that of the commercial ceramic membrane.

## 1. Introduction

Anodic porous alumina films, which are typical self-ordered nanoporous materials, formed by the anodizing of aluminum (Al) in an appropriate acidic or alkaline solution, have attracted considerable attention due to their unique solid geometry, consisting of a so-called honeycomb structure with dimensions ranging from the submicron to nanometer scales [1,2,3,4,5,6,7,8,9,10]. These nanoporous films have been widely used in various applications such as filters, catalyst supports, and templates, as well as electronic, magnetic, and optical devices [11,12,13,14,15]. However, commercially available anodic porous alumina membranes, e.g., Anodisc^®^ (Maidstone, England) [11], have insufficient pore arrangement and low chemical resistance because the porous alumina films obtained using typical anodizing processes are amorphous. To expand the application of anodic porous alumina with ordered pore arrays, it is necessary to control the cell dimensions (e.g., pore interval, pore diameter, and pore depth) and to enhance the chemical resistance of alumina membranes in extreme environments such as high temperature, high vapor pressure, and high-concentration acid/base solutions. If the fabrication of α-alumina membranes with high chemical resistance can be achieved, such membranes can be utilized for filtration under aggressive environments and can possibly be recycled. In addition, the fabrication of α-alumina membranes with an extensive range of pore diameters is also essential.

Several fundamental studies have been reported on the fabrication of crystalline anodic porous alumina aimed at improving the chemical properties of α-alumina membranes using a combination of anodizing and heat treatment processes [16,17]. Mardilovich *et al*. [18] and Ozao *et al*. [19] investigated the transition temperatures and crystal structures of anodic alumina formed in sulfuric acid and oxalic acid using thermogravimetry-differential thermal analysis (TG-DTA) and X-ray diffraction (XRD). As reported in previous studies, porous anodic alumina film have been crystallized to γ- and α-alumina above 900 °C and 1200 °C, respectively. Since the 2000s, the crystallization of free-standing alumina membranes to the α-phase has also been attempted; however, curling and cracking as a result of thermal deformation caused by the change in density as well as decomposition and desorption of electrolyte anions from the outer anion-incorporated layer in the cell wall were unavoidable during the transition to crystalline alumina in addition to the desorption of bound water at lower temperatures [20]. The density of amorphous anodic alumina formed in oxalic acid has been reported to be approximately 3.1 g·cm^−3^ [21], which represents a 23% volume shrinkage after crystallization because the density of α alumina is 4.0 g·cm^−3^.

In 2012, Chang *et al.* [22] fabricated a square-shaped, flat, α-alumina membrane by selectively removing outer phosphate-containing layers from the unit cells of an anodic Al oxide membrane after hydrothermal treatment. They used alumina membranes with a large cell diameter of approximately 500 nm that were formed at a high voltage of 205 V in phosphoric acid containing aluminum oxalate as the starting material. After removing the outer anion-containing layers, they found that the remnant inner layer was composed of relatively pure and dense alumina with good strength. However, the size of the membrane was less than 1 cm^2^. It is worth noting that the deformation of the membrane was effectively suppressed using an anodic membrane composed of only a pure inner alumina layer because deformation is strongly influenced by the decomposition and desorption of the electrolyte anions. Ono and Masuko fabricated an alumina film in phosphoric acid and found that the ratio of the thickness of the pure alumina layer to the thickness of the entire cell wall of the anodic film was approximately 25% [23,24,25], and the alumina layer was easily distinguishable as a crystallized layer under electron irradiation [23]. The membrane prepared by Chang *et al.* appears to have a cell wall thickness of 50–60 nm and a pore size of 450 nm. Their membrane could have sufficient strength for thermal deformation and practical usage. Nevertheless, if one prepares an alumina membrane by removing the anion-containing layer of the film formed in oxalic acid at 40 V with a cell size of 100 nm, the pure alumina layer of the film accounts for only 10% or 3 nm of the thickness [23,24,25]. Therefore, maintaining sufficient mechanical strength has been difficult to achieve for use as a filtration membrane after the removal of anion-incorporated layers, except for films formed in phosphoric acid at high voltage. Thus, the establishment of a fabrication process that provides practically usable, large, flat α-alumina membranes with a wide range of pore diameters has not yet been accomplished.

To detach the anodic film from the Al substrate, immersion in mercury (II) chloride (HgCl_2_) [13], copper (II) chloride (CuCl_2_)-hydrochloric acid [18,19], or iodine-methanol [26] solutions, thinning of the barrier layer using stepwise voltage drops, which is referred to as the current recovery method [11,27], and anodic polarization in ethanol containing perchloric acid [28] have been reported. Most of these film detachment methods for the fabrication of alumina membranes involve the removal of the Al substrate via chemical dissolution. However, the through-hole post-treatment methods that are based on the chemical dissolution of thick barrier layers are time consuming and lead to an excess thinning of the cell walls, which is one of the main reasons for membrane cracking during heating [29,30].

Previously, we investigated the effects of the microstructure of Al substrates on the formation of nanoporous α-alumina membranes by determining the grain size and crystal orientation before and after preheat (recrystallization) treatment [31]. The pore regularity and thermal stability of the membranes were improved when slow heating and, in particular, cooling rates were used during the recrystallization of the Al substrate. Moreover, the optimized anodizing conditions and detachment method for suppressing thermal deformation, such as curving and cracking, during heat treatment of the alumina membrane were identified [29,30]. As a result, the fabrication of a crack-free and flat α-alumina membrane with a diameter of 25 mm, a thickness of 50 μm, and a pore diameter of approximately 50 nm was fabricated using the optimized anodizing conditions in oxalic acid at 40 V, followed by subsequent detachment from the substrate and heat treatment. In the present study, the optimized conditions were further investigated to produce well-controlled nanoporous α-alumina membrane structures with pore diameters tunable over a wide range of 60–350 nm. The pore diameter was tuned by changing the anodizing conditions, including the electrolyte species and formation voltage, and the crystallization process of the porous anodic alumina was examined in detail. To evaluate the potential of these membranes in practical applications, hardness, chemical resistance, and filtration properties of the fabricated α-alumina membranes were examined.

## 2. Results and Discussion

### 2.1. Fabrication of Porous Anodic Alumina Membranes

**Figure 1:**
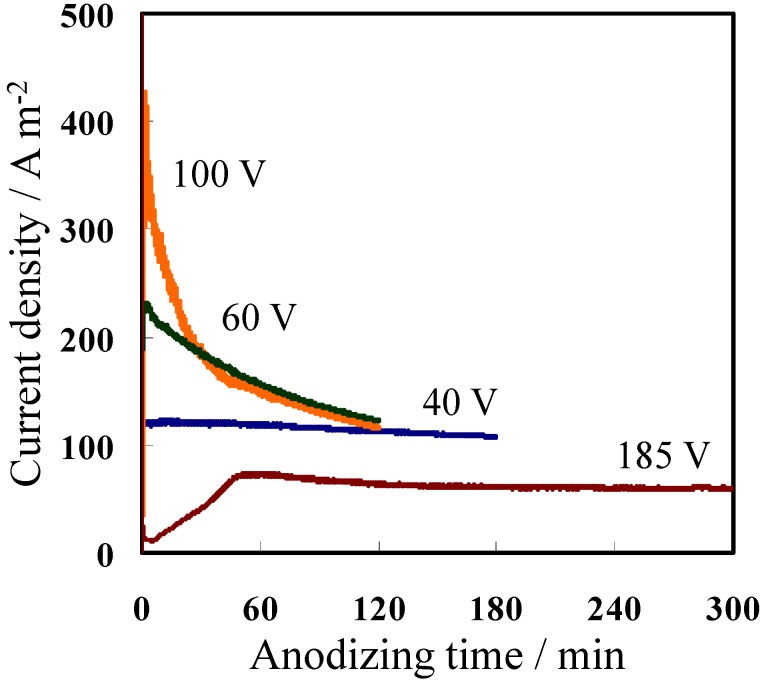
Current–time curves during the second anodizing step using the different voltages listed in Table 1.

Figure 1 shows the current–time (I–t) curves obtained during the second anodizing step using different formation voltages and electrolytes listed in Table 1. To prepare anodic oxide films with a thickness of approximately 50 μm, the anodizing time was adjusted for individual conditions based on the total electricity (1.3 MC·m^−2^) consumed during anodizing at 40 V in oxalic acid. The current density during anodizing at both 40 V and 185 V was stable. When anodizing at 185 V in phosphoric acid, the temperature was controlled at 15 °C to avoid burning during the first 1 h while the current density was kept constant. Meanwhile, the current density during anodizing at 60 V and 100 V was high, particularly during the initial stage, but eventually decreased to 120–140 Am^−2^.

**Table 1:** Anodizing conditions employed for the fabrication of alumina membranes with different pore diameters.

Electrolytes	Temperature (°C)	Formation Voltage (V)		Anodizing Time (min)
		1st	2nd	
0.3 mol·dm^−3^ oxalic acid	30	40	40	180
0.3 mol·dm^−3^ oxalic acid	20	55	60	120
0.3 mol·dm^−3^ oxalic acid–0.2 mol·dm^−3^ phosphoric acid	25	100	100	120
0.2 mol·dm^−3^ phosphoric acid	5–15	185	185	300

**Figure 2:**
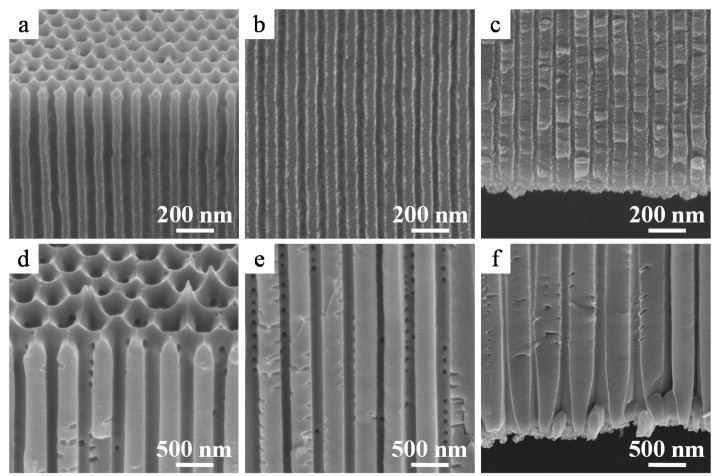
Cross-sectional scanning electron microscopy (SEM) images of the as-detached amorphous alumina membranes formed in (**a**–**c**) oxalic acid at 40 V and (**d**–**f**) phosphoric acid at 185 V. (**a**,**d**) Top, (**b**,**e**) middle, and (**c**,**f**) bottom portions of the alumina membranes.

Cross-sectional scanning electron microscopy (SEM) images of the anodic film formed in oxalic acid at 40 V after detachment from the Al substrate are shown in Figure 2a–c. The anodic film was successfully detached from the Al substrate using anodic polarization in a perchloric acid-ethanol mixture. The pore diameter was substantially larger at the top (Figure 2a) than in the middle (Figure 2b) and the bottom (Figure 2c) parts of the membrane. This pore enlargement is caused by cell wall dissolution, *i.e.*, pore-widening because of prolonged immersion in the acidic electrolyte during anodizing for 3 h. The barrier layer of the bottom part of the films formed at 40 V and 60 V were actually broken and removed because of aggressive dissolution of the substrate following the sudden application of a high anodic bias. Figure 2d–f show the cross-sectional images of the alumina film formed in phosphoric acid at 185 V after detachment from the Al substrate. Once again, the pore diameter at the top was more than twice of that observed at the bottom portion of the film. In addition, branched pores detected in the bottom portion (Figure 2f) were formed during a stepwise voltage reduction to 50 V. Thus, a free-standing film with a pore diameter of 206 nm at the top surface (Figure 2d) was obtained using the current recovery (stepwise voltage reduction) method, even when the formation voltage was as high as 185 V. Interestingly, small lateral holes were detected in the cell wall (see the arrows in Figure 2d). It was assumed that these holes formed due to the presence of copper in the Al substrate [32], which led to breakdown between the pore bases and triple points of the cells [23,33]. This assumption was confirmed when a membrane fabricated using an Al substrate containing only 25 ppm copper was found to have few lateral holes in its cell wall.

**Figure 3:**
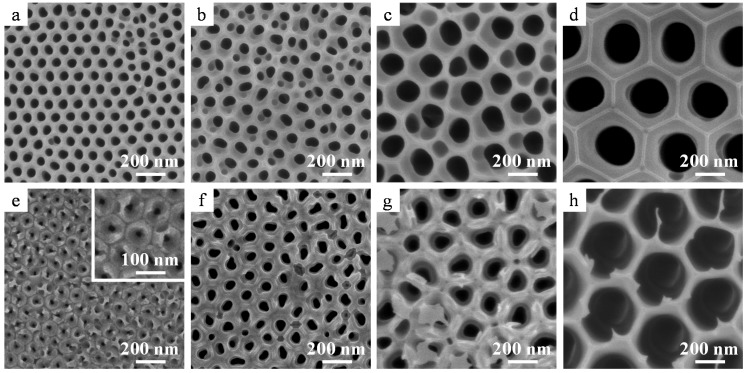
SEM images of (**a**–**d**) top and (**e**–**h**) rear surfaces of amorphous alumina membranes formed via anodizing at (**a**,**e**) 40 V, (**b**,**f**) 60 V, (**c**,**g**) 100 V, and (**d**,**h**) 185 V. The inset indicates a high magnification image of the rear surface. Through-hole treatment was conducted in 5 wt% phosphoric acid at 30 °C.

Figure 3 shows the top and rear surface images of amorphous alumina membranes with different pore diameters formed at 40 V, 60 V, 100 V, and 185 V after through-hole formation via dipping in phosphoric acid for 5, 30, 60, and 120 min, respectively. The protruding hexagonal cell junctions observed in Figure 3d were part of a chemically resistant anion-free layer of the film formed in phosphoric acid [23]. The pore diameters of the top surfaces after through-hole formation via chemical etching were 62, 79, 164, and 282 nm for the films formed at 40 V, 60 V, 100 V, and 185 V, respectively, whereas those of the rear surfaces were 20, 60, 97, and 357 nm, respectively. The pore diameter of the rear surface of the film formed at 40 V was smaller than that expected from the cross sectional image shown in Figure 2c because the barrier layer was only partially removed. In addition, the rear surface of the film formed at 185 V appeared wider because the cell wall at the tip of the rear surface was thinned owing to the voltage drop during the stepwise current reduction (current recovery) method (see section 3.1). Thus, the apparent pore diameter of the rear surface after through-hole formation observed using SEM is not the same as the real pore diameter in some cases.

### 2.2. Crystallization of the Alumina Membranes via Heating

**Figure 4:**
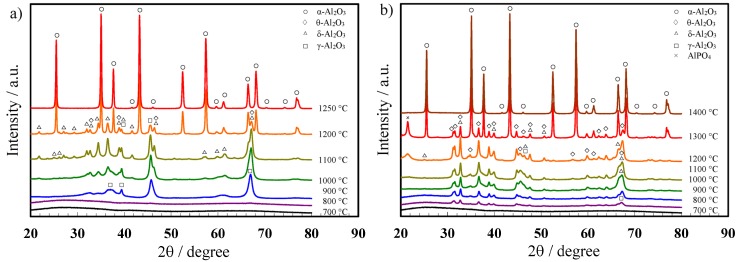
X-ray diffraction patterns for anodic porous alumina films formed in (**a**) oxalic acid at 40 V and (**b**) phosphoric acid at 185 V after heat treatment at various temperatures for 4 h.

Next, to evaluate the effect of the electrolyte species on the crystallization of porous anodic alumina, XRD analyses were conducted for the through-hole membranes after heat treatment at 700–1400 °C for 4 h. The anodic alumina films formed in oxalic acid and phosphoric acid were crystallized to γ-alumina at above 800 °C and then transferred to α-alumina via δ- and θ-alumina. However, the crystallization process to α-alumina was affected by the electrolyte anion species. In the XRD patterns of the films formed in oxalic acid (Figure 4a), only peaks for γ-alumina were detected at 900 °C, whereas peaks for both γ- and δ-alumina were detected at above 1000 °C. In addition, the intensity of these peaks increased with increasing heat treatment temperatures to up to 1100 °C. Moreover, the XRD pattern for the alumina heated at 1200 °C showed distinct peaks for α-alumina in addition to γ- and δ-alumina. Eventually, the anodic alumina was completely crystallized to the α phase by heat treatment at 1250 °C for 4 h.

For the anodic alumina films formed in phosphoric acid, peaks for δ- and θ-alumina appeared together with γ-alumina in the XRD patterns below 1200 °C (Figure 4b). In addition, the peak for AlPO_4_ was detected at 21° in the XRD patterns for the alumina films heated at 1200 °C and 1300 °C, although this peak disappeared after heat treatment at 1400 °C due to the thermal decomposition of AlPO_4_. Notably, the transition temperature for the anodic alumina formed in phosphoric acid to the α phase was 100 °C higher than that for the alumina film formed in oxalic acid, presumably because of the presence of PO_4_^3−^ anions in the film.

Furthermore, thermogravimetry-differential thermal analysis (TG-DTA) was performed to evaluate the structures of the fabricated amorphous films. For the films formed in oxalic acid, two main desorption peaks were observed in the TG-DTA weight loss curves [29,30]. The initial weight loss of 1.7% from room temperature to approximately 600 °C indicated dehydration corresponding to the loss of both physisorbed and chemically bound water. From 600 to 1200 °C, a weight loss of approximately 5% was largely attributed to the release of CO and CO_2_ following the decomposition of oxalate anions at 880 °C. The exothermic peak indicating crystallization to γ-alumina has been observed previously [30]. The peak associated with the transition to α-alumina was detected at 1150 °C in all the TG-DTA curves. The crystallization temperatures for the anodic alumina films determined via XRD (Figure 4a) were therefore in agreement with the TG-DTA results.

For the films formed in phosphoric acid, although no weight loss was detected in the TG curves up to 1500 °C, the DTA curves revealed three exothermic peaks at 879 °C, 1043 °C, and 1336 °C, which corresponded to the γ-, θ-, and α-alumina phases, respectively. The phase changes for these amorphous anodic alumina films with heating occurred in the order of γ- to δ- to θ-, and finally α-alumina, which is similar to that observed for boehmite (Al_2_O_3_·H_2_O) [34]. The minimal weight change during heating for the films formed in phosphoric acid, even after transition to single phase α-alumina, can be explained by the fact that very little bound water was present in the film, and presumably phosphorus oxide, which does not vaporize, remained in the film. Further details are explained elsewhere.

**Figure 5:**
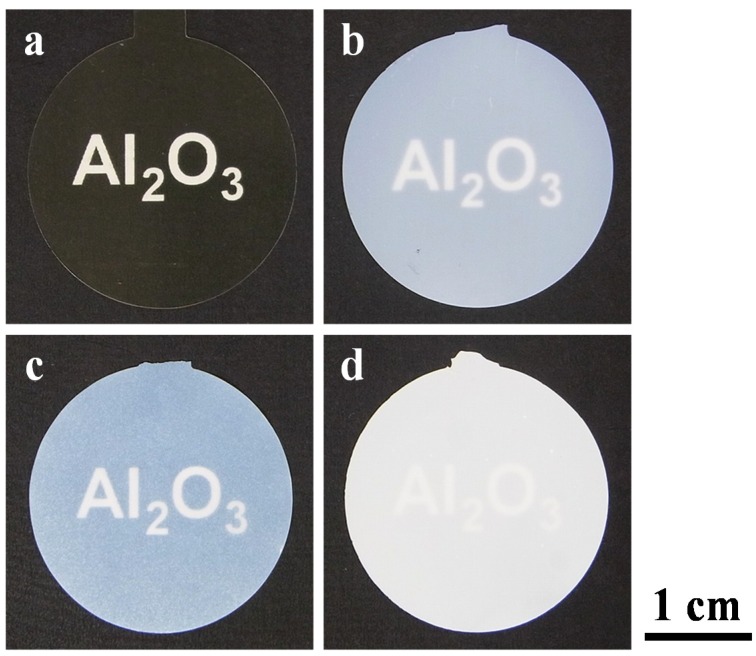
Photographs of (**a**,**b**) amorphous and (**c**,**d**) α-alumina membranes formed in (**a**,**c**) oxalic acid at 40 V and (**b**,**d**) phosphoric acid at 185 V. Through-hole treatment was conducted in phosphoric acid for (**a**,**c**) 5 min and (**b**,**d**) 120 min. Heat treatment was performed at (**c**) 1250 °C and (**d**) 1400 °C for 4 h. Alumina membranes were placed on a black background printed with the word “Al_2_O_3_” in white, and the photographs were taken with a digital camera.

Photographs of the amorphous and α-alumina membranes formed at 40 V and 185 V are shown in Figure 5. Prior to heat treatment, the alumina membranes formed in oxalic acid and phosphoric acid were pale yellow and transparent (Figure 5a) and milky white and translucent (Figure 5b), respectively. The film formed in oxalic acid turned milky white and translucent after heat treatment at 1250 °C (Figure 5c), whereas the membrane formed in phosphoric acid turned whiter and more opaque after heat treatment at 1400 °C (Figure 5d). By adjusting the chemical dissolution time on the basis of our previous study [30], it was possible to obtain flat, warp/crack-free α-alumina membranes, regardless of the formation voltage. The diameters (ϕ = 25 mm) of the membranes formed in oxalic acid at 40 V and phosphoric acid at 185 V shrunk approximately to 3%–4% and 2%–3%, respectively, after transition to α-alumina. This shrinkage occurred because of an increase in the density of alumina after crystallization, dehydration, and desorption of the electrolyte anions, as described below.

### 2.3. Nanostructure of the α-Alumina Membranes

**Figure 6:**
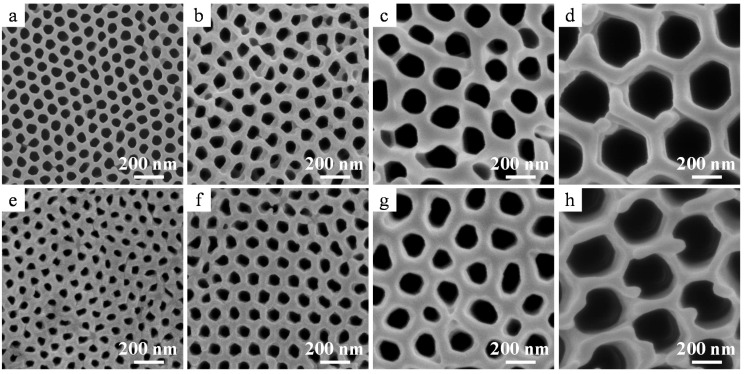
SEM images of the (**a**–**d**) top and (**e**–**h**) rear surfaces of the α-alumina membranes formed at (**a**,**e**) 40 V, (**b**,**f**) 60 V, (**c**,**g**) 100 V, and (**d**,**h**) 185 V. Heat treatment was conducted at (**a**,**b**,**e**,**f**) 1250 °C or (**c**,**d**,**g**,**h**) 1400 °C for 4 h.

Both the top (Figure 6a–d) and rear surfaces (Figure 6e–h) of the α-alumina membranes appeared flat and uniform. All the uneven cell morphologies observed for amorphous alumina films before heat treatment on both the top and rear surfaces (Figure 3) were flattened after heat treatment owing to the sintering of the alumina (Figure 6a–d). Simultaneously, the residual barrier layers observed in Figure 2e,f were absorbed into the pore walls due to sintering (Figure 6e,f), and the pore diameters at both the surfaces were similar. Further, the branched nanoporous layer formed on the rear surface as a result of the stepwise voltage reduction also disappeared during heat treatment (Figure 6g,h).

**Figure 7:**
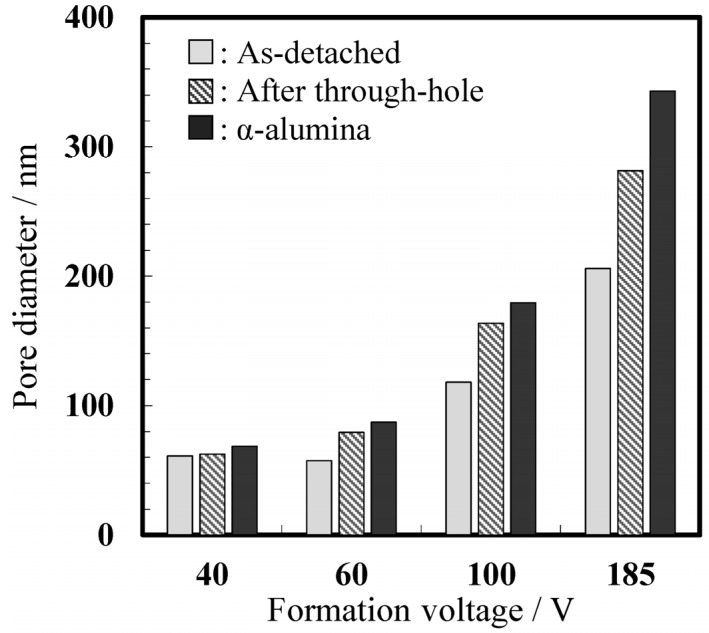
Changes in the pore diameters of the top surfaces of alumina membranes after through-hole and subsequent heat treatment. The chemical dissolution of the alumina membranes formed at 40, 60, 100, and 185 V was performed in 5 wt% phosphoric acid at 30 °C for 5, 30, 60, and 120 min, respectively.

The pore diameters of the top surfaces of the as-formed and chemically etched alumina membranes prepared at different voltages before and after crystallization are summarized in Figure 7. Table S1 provides detailed information about the pore diameters of the obtained membranes. After detachment from the substrate and chemical dissolution for through-hole treatment, the average pore diameter of the top surface of the amorphous membrane film formed in oxalic acid at 40 V was 62 nm, which increased to 69 nm after crystallization to the α phase following heat treatment at 1250 °C for 4 h (Figure 6a and Figure 7). This change represents an approximately 9% increase in the pore size. In addition, the pore diameters at the top surfaces of the membranes formed at 60 V and 185 V increased from 79 to 87 nm (Figure 6b) and 282 to 343 nm (Figure 6d), respectively, which are equivalent to 10% and 20% increase after crystallization. However, it should be noted that the pore diameter of the top surface is always larger than that of the rear surface because of chemical dissolution in the electrolyte during anodizing. In fact, cross sections of the middle layer of the membrane indicated that the average pore diameter of the α-alumina membranes formed in oxalic acid at 40 V and phosphoric acid at 185 V were 60 nm and 344 nm, respectively, indicating approximately 14%–20% of increase after crystallization. Thus, the pore diameters of all the alumina membranes were substantially enlarged after crystallization to α-alumina because of the change in the density of the materials. In addition, it should be noted that if the pore diameter can be further enlarged via chemical dissolution, it would be possible to further the pore diameter in the cell diameter range.

It is known that the density of amorphous alumina formed at 100 A·m^−2^, which corresponds to a formation voltage of approximately 40 V in oxalic acid, is 3.1 g·cm^−3^ [21], while the density of α-alumina is approximately 4.0 gppr^−3^. Therefore, a 23% volume shrinkage must occur after crystallization to α-alumina. In the present study, the membrane diameter was approximately 3% smaller after crystallization, which corresponds to a 6% shrinkage in oxide volume. Hence, a cell volume shrinkage of approximately 17% should have occurred. Therefore, a pore diameter enlargement of approximately 20% is in good agreement with the predicted results for the membranes formed at both 40 V and 185 V.

### 2.4. Properties of the α-Alumina Membranes

#### 2.4.1. Chemical Resistance

**Figure 8:**
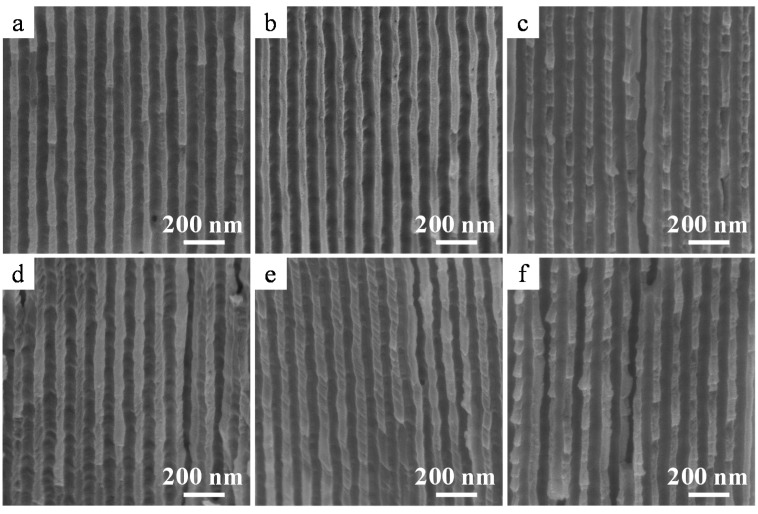
Cross-sectional SEM images of α-alumina membranes formed in 0.3 mol·dm^−3^ oxalic acid at 40 V after chemical resistance tests. (**a**) As-heated at 1250 °C for 4 h and after immersion in (**b**) concentrated H_2_SO_4_; (**c**) concentrated H_3_PO_4_; (**d**) 10 mol·dm^−3^ KOH; (**e**) 10 mol·dm^−3^ NaOH at room temperature for 2500 h; and (**f**) pressurized water vapor at 300 °C for 720 h.

Figure 8 shows the cross-sectional SEM images of α-alumina membranes formed in oxalic acid at 40 V after immersion in concentrated acidic or alkaline solutions for 2500 h (3 months) and in 8.6 MPa pressurized water vapor at 300 °C for 720 h (1 month). As can be seen in Figure 8b–f, few changes were observed in α-alumina membranes, and they maintained the cylindrical pore structure obtained after crystallization (Figure 8a). The cell walls of the α-alumina membranes were composed of node-like grains with sizes similar to those of the cell walls. These node-like grains are crystallites of α alumina, although the crystal grain sizes of the membranes were found to be approximately 100 μm [31]. The α-alumina membranes immersed in various concentrated acidic (Figure 8b,c) and alkaline (Figure 8d,e) solutions exhibited no changes after exposure to these aggressive conditions. The α-alumina membrane also did not exhibit any change after immersion in pressurized water vapor at 300 °C for 720 h (Figure 8f). It is well known that as-anodized amorphous membranes can be easily hydrated and sealed with platelet-like hydroxide within several minutes in boiling water [35]. Therefore, the results of the present chemical resistance tests clarified that the α-alumina membranes prepared in this study exhibited high chemical resistance in aggressive environments and were stable over a wide pH range, at high temperatures, and in steam under high-pressure conditions.

#### 2.4.2. Mechanical Properties

**Figure 9:**
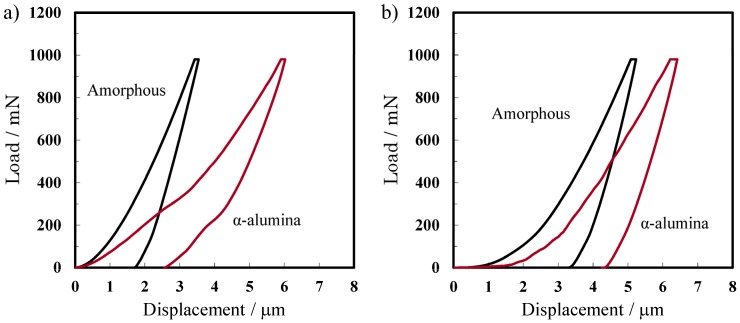
Load-displacement curves for the amorphous and α-alumina membranes formed in (**a**) oxalic acid at 40 V and (**b**) phosphoric acid at 185 V. Nanoindentation was performed with a 980 mN (100 gf) load and a dwell time of 15 s.

Figure 9 presents the load-displacement curves for the amorphous and crystalline alumina membranes during nanoindentation at 980 mN. The results reveal that the amount of displacement for the α-alumina membrane prepared in oxalic acid at 40 V increased up to 1.7 times than that of the corresponding amorphous alumina membrane at the peak load. For the α-alumina membrane prepared at 185 V, the displacement increased by a factor of 1.2 after transition from the amorphous to the α phase. The Young’s modulus and hardness of each of the amorphous and α-alumina membranes, along with their porosities estimated form surface pore diameters, are summarized in Table 2. The Young’s modulus, which represents the rigidity of the material, and the hardness, decreased after crystallization to α-alumina for the amorphous membranes prepared at both 40 V and 185 V. These results are not in agreement with those previously reported by McQuaig Jr *et al.* [20], who reported that the hardness of anodic alumina membranes increased after crystallization at 1200 °C. In the present study, the hardness of the amorphous membrane prepared at 40 V was 444 and declined to 405 at 1100 °C, 430 at 1200 °C, and finally 348 at 1250 °C, *i.e.*, the transition temperature to a single α-alumina phase. The discrepancy between the present results and the previously reported data may be due to a difference in the crystal grain structure of α-alumina [31] and the presence of nodular crystallites observed in the membrane cross sections shown in Figure 8, the formation of which was highly sensitive to the heating conditions. Further details will be reported elsewhere.

**Table 2:** Relationship between the porosity, hardness, and Young’s modulus of the alumina membranes formed via anodizing at different voltages. Anodizing was conducted under the same conditions as those described in Table 1.

Formation Voltages	40 V		185 V	
	Amorphous	α-alumina	Amorphous	α-alumina
Young’s modulus (GPa)	47.1 ± 1.4	16.3 ± 0.7	29.0 ± 0.5	19.7 ± 0.9
Hardness (HV)	444 ± 6	349 ± 18	533 ± 10	175 ± 13
Porosity	0.42	0.47	0.45	0.68

Furthermore, it is noteworthy that the changes in hardness indicate a similar tendency for the changes in the porosity of the membranes. Hardness is essentially dependent on the cell wall thickness and the porosity in porous anodic alumina, as reported by Fukuda *et al.* [36]. Because the cell wall thickness decreased and the porosity increased after transition to the α-phase, the changes in the hardness shown in Table 2 are reasonable.

#### 2.4.3. Filtration Properties

**Figure 10:**
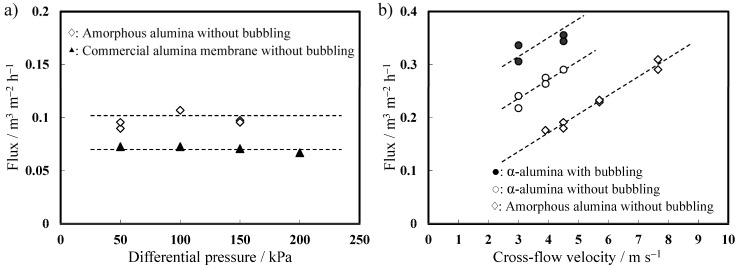
Relationship between the flux and (**a**) differential pressure and (**b**) cross-flow velocity of alumina membranes formed by anodizing in 0.2 mol·dm^−3^ phosphoric acid at 185 V when compared with those of commercially available alumina membrane. The permeation test was conducted using an 8000 mg·L^−1^ mixed liquor suspended solids solution in the cross-flow mode.

Figure 10 shows the filtration properties of the amorphous and α-alumina membranes formed in phosphoric acid at 185 V in comparison with the commercially availabe Anodisc^®^ membrane evaluated at various differential pressures and various surface flow velocities using an 8000 mg·L^−1^ solution of mixed liquor suspended solids (MLSS) at 20 °C. The flux was independent of differential pressure in both membranes of the commercial membrane and the amorphous membranes; however, the latter’s flux was considerably higher (Figure 10a). The flux linearly increases with increasing cross-flow velocity (Figure 10b). Even if the cross-flow velocity is zero, the flux does not go down to zero when a specific differential pressure is maintained between the inlet and outlet sides of a membrane to push a liquid through the membrane. When the cross-flow velocity was 4.5 m·s^−1^, the average flux of the amorphous and α-alumina membranes without bubbling was 0.18 m^3^·m^−2^·h^−1^ and 0.29 m^3^· m^−2^·h^−1^, respectively. The increase in flux was mainly due to the enlargement of the pore diameter with crystallization. The flux of the α-alumina membrane under bubbling conditions increased by approximately 20% compared to that without bubbling under the same cross-flow velocity owing to a reduction in fouling.

Thus, the level of flux for the amorphous and α-alumina membranes was significantly higher than that of the commercial alumina membrane. Furthermore, the α-alumina membrane should be reusable after the removal of any fouling and/or formed biofilm using an acid or alkali treatment because it is entirely insoluble under such conditions. Thus, the results of the filtration test confirmed that the as-formed anodic α-alumina membrane has significant potential for commercial application.

## 3. Experimental Section

### 3.1. Preparation of the Anodic Porous Alumina Membranes

**Figure 11:**
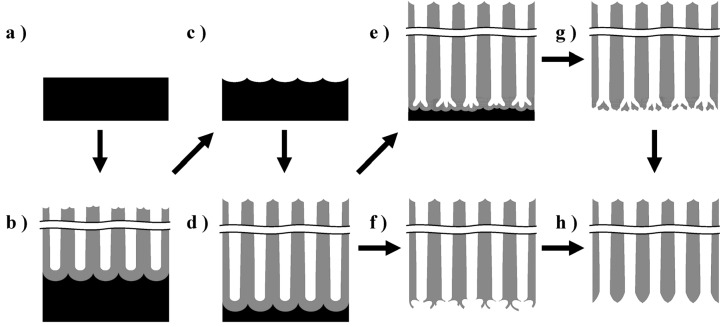
Schematic of the fabrication process of anodic porous alumina membranes (**a**) Electropolishing; (**b**) First anodizing step; (**c**) Removal of anodic film; (**d**) Second anodizing step; (**e**) Barrier layer thickness reduction for the films formed at voltages higher than 100 V using a stepwise voltage reduction; (**f**) and (**g**) Detachment of the anodic film; (**h**) Chemical etching in 5 wt% phosphoric acid at 30 °C.

The anodic porous alumina membranes were prepared using the multistep anodizing process described previously [31]. Figure 11 presents a schematic of the fabrication process for the anodic porous alumina membranes. A high-purity (99.99%) Al sheet supplied by Toyo aluminium KK with a thickness of 0.35 mm was cut into 25-mm-diameter circles with a handle using a wire-electrical discharge machine (FANUC ROBOCUT α-OC). After degreasing the sample in an ultrasonic bath with acetone for 3 min, electropolishing was performed below 10 °C for 2 min in a 1:4 (by volume) mixed solution of perchloric acid (60%) and ethanol (99.5%) at a constant current of 1500 A·m^−2^ (Figure 11a).

The anodizing conditions for the preparation of membranes with different pore diameters are listed in Table 1. The first anodizing step was performed under a constant voltage for 1 h (Figure 11b). The obtained oxide film was then immersed in a boiling mixed solution of 2 wt% chromic acid and 6 wt% phosphoric acid for 10 min (Figure 11c). The second anodizing step was performed under the same conditions as those used in the first step (Figure 11d). This process significantly improved the pore arrangement and prevented numerous small pore initiations at the film surface, as reported by Masuda *et al.* [5]. The film thickness was adjusted to approximately 50 μm based on the total quantity of electricity consumed during anodizing at 40 V in oxalic acid. For the membrane prepared at 60 V in oxalic acid, the first anodizing was performed at 55 V to ensure that the ratio of pore generation sites in the first and second anodizing steps was 1:1. For the anodizing processes in phosphoric acid, the temperature of the electrolyte was regulated at 5 °C during the first 10 min and then gradually increased to 15 °C to avoid local current concentration “burning” [37].

After the second anodizing step, the anodized Al was pre-heated at 500 °C for 2 h to impart acid resistance to the anodic film, as reported previously [38]. Prior to detachment, the films formed at 40 V and 60 V were anodized again in 0.2 mol·dm^−3^ phosphoric acid for 1 min to grow an unheated new oxide layer on the substrate under the heated oxide film (Figure 11d). For the anodic oxide films formed at voltages higher than 100 V, a stepwise voltage reduction from the formation voltage (*i.e.*, 100 and 185 V) to 50 V was performed in the same electrolyte used for the second anodizing step to reduce the barrier layer thickness (Figure 11e). To detach the anodic films from the substrates, anodic polarization was applied in a mixed solution of perchloric acid and ethanol, *i.e.*, an electropolishing electrolyte, for 1 min (Figure 11f,g) at a voltage of 10 V higher than the formation voltage, as described previously [28,31]. Finally, the detached anodic films, *i.e.*, alumina membranes, were chemically etched in 5 wt% phosphoric acid at 30 °C to remove any residual barrier layer, for through-hole formation, and to adjust the pore diameter (Figure 11g).

### 3.2. Crystallization of the Alumina Membrane via Heat Treatment

After through-hole treatment, the alumina membranes were placed between two ceramic cordierite-mullite plates, which were used as the restriction load, and then heated in a furnace (ADVANTECH FUH612DA) in an air atmospheric using a controlled temperature program. The temperature was gradually increased from room temperature up to 1400 °C at a heating rate of 25–200 °C·h^−1^ to prevent thermal cracking by applying a lower heating rate around the transition temperature of crystal phase. The specimens were held at desired temperature for 4 h and then cooled to room temperature in the furnace at a cooling rate of 50 °C·h^−1^. Additional information for a controlled temperature program is provided in Figure S1.

### 3.3. Characterization of the α-Alumina Membranes

The nanoporous structures of the anodic alumina membranes were evaluated using field-emission scanning electron microscopy (FE-SEM, JEOL JSM-6701F). The average pore diameters were determined from the SEM images of the top and bottom surfaces using the image analysis software in the public domain, Image J. The average pore size distribution and maximum pore size of the alumina membranes were estimated via pore size distribution analysis (PMI CFP-1500 AELT). Thermogravimetry-differential thermal analysis (TG-DTA, RIGAKU TG8120) was performed using 10 mg of powdered sample placed in an alumina pan in air with using a heating rate of 10 °C·min^−1^ up to 1500 °C. The crystal structures of the heated alumina films were evaluated using XRD analysis (Bruker AXS MXP-18AHF22) with Cu-K α radiation generated at 45 kV and 300 mA.

To investigate the chemical resistance of the α-alumina membranes to acidic and alkaline solutions, they were each immersed separately in 18 mol·dm^−3^ sulfuric acid, 15 mol·dm^−3^ phosphoric acid, 10 mol·dm^−3^ sodium hydroxide, and 10 mol·dm^−3^ potassium hydroxide, respectively for 2500 h (3 months) at 20 °C. Their chemical resistance was also evaluated in steam under a pressure of 8.6 MPa at 300 °C for 720 h (1 month). After each chemical resistance test, the nanoporous structure of the films was investigated using SEM.

To evaluate the hardness of the alumina membranes, nanoindentation tests were performed using a hardness tester (ERIONIX ent-1100a) at five locations on each membrane with a dwell time of 15 s. The porosities α of the porous membranes were calculated using the following equation:

α = (*d*_pore_/*d*_cell_)^2^ (1)

where *d*_pore_ is the surface pore diameter estimated from the circumference using the ImageJ analysis software and *d*_cell_ is the cell diameter determined via SEM observation of the top surface.

Filtration experiments were conducted using a laboratory-scale cross-flow membrane filtration unit (See Figure S2). The filtration properties of the α-alumina membranes were evaluated at various surface flow velocities using MLSS with a concentration of 8000 mg·L^−1^ at 20 °C. For the flux measurements, the filtrate water was collected for a specific period of time during the filtration run. Air bubbling was provided to the membrane surface by an air injector as shown in Figure S2. The effects of air bubbling on filtration properties were evaluated to increase flux.

## 4. Conclusions

(1) Nanoporous α-alumina membranes with tunable pore diameters ranging from 60 to 350 nm were successfully fabricated using optimized conditions for anodizing, detachment, and heat treatment. The pore diameter and cell diameter were enlarged and shrunk by approximately 20% and 3%, respectively, following crystallization to α-alumina, which is in accordance with the 23% volume shrinkage associated with the change in density that occurs with the amorphous to α-alumina transition. Nevertheless, the flat α-alumina membranes of 25 mm diameter without the thermal deformation were obtained. The following points are considered as the major factors for successful synthesis of reasonably sized α-alumina membranes. (I) Two-step anodizing significantly improved the pore arrangement and prevented numerous small pore initiations at the film surface, resulting in the improvement of the membranes’ structural symmetry; (II) By shortening of the processing time of detachment and through-hole methods based on the chemical dissolution of barrier layers, an excess thinning of the cell walls was kept to the minimum. Because hardness is essentially dependent on the cell wall thickness and the porosity in porous anodic alumina, the adjustment of the chemical dissolution time is absolutely imperative; (III) To prevent thermal cracking, the alumina membranes were placed between two ceramic cordierite-Mullite plates and heated using a controlled temperature program by applying a lower heating rate around the transition temperature of crystal phase.

(2) The nanoindentation hardness of the alumina membrane increased by 30% as the heating temperature increased to 1200 °C. However, the Young’s modulus and hardness of the single phase α-alumina membrane formed by heat treatment at 1250 °C notably decreased. This change is attributed to the nodular crystallites structure of the cell wall and a significant increase in porosity.

(3) The obtained α-alumina membranes have high chemical resistance to concentrated acidic or alkaline solutions and high temperature steam under pressure.

(4) Importantly, the α-alumina membrane formed in phosphoric acid at 185 V exhibited a level of flux significantly higher than that of commercial ceramic membrane. The α-alumina membrane should also be reusable because any fouling and/or biofilms formed on the membrane can be removed by acid or alkali treatment.

## Supplementary Materials

Supplementary materials can be accessed at: http://www.mdpi.com/1996-1944/8/3/1350/s1.

## References

[1] Keller F., Hunter M.S., Robinson D.L. (1953). Structural features of oxide coatings on aluminum. J. Electrochem. Soc..

[2] Diggle J.W., Downie T.C., Goulding C.W. (1969). Anodic oxide films on aluminum. Chem. Rev..

[3] O’Sullivan J.P., Wood G.C. (1970). The morphology and mechanism of formation of porous anodic films on aluminium. Proc. Roy. Soc. Lond..

[4] Dekker A., Middelhoek A. (1970). Transport numbers and the structure of porous anodic films on aluminum. J. Electrochem. Soc..

[5] Masuda H., Fukuda K. (1995). Ordered metal nanohole arrays made by a two-step replication of honeycomb structures of anodic alumina. Science.

[6] Masuda H., Satoh M. (1996). Fabrication of gold nanodot array using anodic porous alumina as an evaporation mask. Jpn. J. Appl. Phys..

[7] Masuda H., Hasegawa F., Ono S. (1997). Self-ordering of cell arrangement of anodic porous alumina formed in sulfuric acid solution. J. Electrochem. Soc..

[8] Masuda H., Yada K., Osaka A. (1998). Self-ordering of cell configuration of anodic porous alumina with large-size pores in phosphoric acid solution. Jpn. J. Appl. Phys..

[9] Ono S., Saito M., Ishiguro M., Asoh H. (2004). Controlling factor of self-ordering of anodic porous Alumina. J. Electrochem. Soc..

[10] Ono S., Saito M., Asoh H. (2005). Self-ordering of anodic porous alumina formed in organic acid electrolytes. Electrochim. Acta.

[11] Furneaux R.C., Rigby W.R., Davidson A.P. (1989). The formation of controlled-porosity membranes from anodically oxidized aluminium. Nature.

[12] Martin C.R. (1994). Nanomaterials: A membrane-based synthetic approach. Science.

[13] Li A.P., Müller F., Birner A., Nielsch K., Gösele U. (1998). Self-organized formation of hexagonal pore arrays in anodic alumina. J. Appl. Phys..

[14] Kouklin N., Menon L., Wong A.Z., Thompson D.W., Woollam J.A., Williams P.F., Bandyopadhyay S. (2001). Giant photoresistivity and optically controlled switching in self-assembled nanowires. Appl. Phys. Lett..

[15] Yanagishita T., Tomabechi Y., Nishio K., Masuda H. (2004). Preparation of monodisperse SiO_2_ nanoparticles by membrane emulsification using ideally ordered anodic porous alumina. Langmuir.

[16] Fernández-Romero L., Montero-Moreno J.M., Pellicer E., Peiró F., Cornet A., Morante J.R., Sarret M., Müller C. (2008). Assessment of the thermal stability of anodic alumina membranes at high temperatures. Mater. Chem. Phys..

[17] Choudhari K.S., Sudheendra P., Udayashankar N.K. (2012). Fabrication and high-temperature structural characterization study of porous anodic alumina membranes. J. Porous Mat..

[18] Mardilovich P.P., Govyadinov A.N., Mukhurov N.I., Rzhevskii A.M., Paterson R. (1995). Assessment of the thermal stability of anodic alumina membranes at high temperatures. J. Membr. Sci..

[19] Ozao R., Yoshida H., Ichikawa Y., Inada T., Ochiai M. (2001). Crystallization of anodic alumina membranes studied by simultaneous TG-DTA/FTIR. J. Therm. Anal. Calorim..

[20] McQuaing M.K., Toro A., Geertruyden W.V., Misiolek W.Z. (2011). The effect of high temperature heat treatment on the structure and properties of anodic aluminum oxide. J. Mater. Sci..

[21] Ebihara K., Takahashi H., Nagayama M. (1983). Structure and density of anodic oxide films formed on aluminum in oxalic acid solutions. J. Surf. Finish. Soc. Jpn..

[22] Chang Y., Ling Z., Liu Y., Hu X., Li Y. (2012). A simple method for fabrication of highly ordered porous α-alumina ceramic membranes. J. Mater. Chem..

[23] Ono S., Masuko N. (1992). The duplex structure of cell walls of porous anodic films formed on aluminum. Corros. Sci..

[24] Ono S., Masuko N. (1993). Dissolution behavior of the barrier layer of porous anodic films formed on aluminum studied by pore-filling technique. J. Jpn. Inst. Light Met..

[25] Ono S., Masuko N. (1993). Electron microscopic study of the structure and dissolution behavior of porous anodic films formed on aluminum. J. Jpn. Inst. Light Met..

[26] Kirchner A., MacKenzie K.J.D., Brown I.W.M., Kemmitt T., Bowden M.E. (2007). Structural characterisation of heat-treated anodic alumina membranes prepared using a simplified fabrication process. J. Membr. Sci..

[27] Takahashi H., Nagayama M., Akahori H., Kitahara A. (1973). Electron-microscopy of porous anodic oxide films on aluminium by ultra-thin sectioning technique part 1. The structural change of the film during the current recovery period. J. Electron Microsc..

[28] Yuan J.H., He F.Y., Sun D.C., Xia X.H. (2004). A simple method for preparation of through-hole porous anodic alumina membrane. Chem. Mater..

[29] Ono S., Nakamura M., Masuda T., Asoh H. (2014). Fabrication of nanoporous crystalline alumina membrane by anodization of aluminum. Mater. Sci. Forum.

[30] Masuda T., Asoh H., Haraguchi S., Ono S. (2014). Nanoporous α-alumina membrane prepared by anodizing and heat treatment. Electrochemistry.

[31] Rashidi F., Masuda T., Asoh H., Ono S. (2013). Metallographic effects of pure aluminum on properties of nanoporous anodic alumina (NPAA). Surf. Interface Anal..

[32] Molchan I.S., Molchan T.V., Gaponenko N.V., Skeldon P., Thompson G.E. (2010). Impurity-driven defect generation in porous anodic alumina. Electrochem. Commun..

[33] Ono S., Ichinose H., Masuko N. (1991). Defects in porous anodic films formed on high purity aluminum. J. Electrochem. Soc..

[34] Wefers K., Misra C. (1987). Oxides and Hydroxides of Aluminum.

[35] Ono S., Masuko N. (1994). The effect of incorporated anions to the sealing of porous anodic films on aluminum. J. Surf. Finish. Soc. Jpn..

[36] Fukuda Y., Fukushima T. (1980). High temperature hard anodizing with tartaric acid-oxalic acid-triethanolamine bath. Bull. Chem. Soc. Jpn..

[37] Ono S., Saito M., Asoh H. (2004). Self-ordering of anodic porous alumina induced by local current concentration: Burning. Electrochem. Solid State Lett..

[38] Asoh H., Tanabe K., Ono S. (2002). Effect of heat treatment on solubility of anodic porous alumina. J. Surf. Finish. Soc. Jpn..

