# Supplementary material for: Fabrication and Characterization of Single Phase α-Alumina Membranes with Tunable Pore Diameters
Source: Materials (Basel). 2015 Mar 20;8(3):1350–68. doi: 10.3390/ma8031350 (PMC5455454; doi:10.3390/ma8031350)
Supplement: Supplementary file 1 [file materials-08-01350-s001.pdf]

Supplementary Information

Table S1. Changes in the pore diameters of alumina membranes after heat treatment.

Electrolytes	Formation Voltage (V)	Amorphous Alumina Membrane		α-alumina Membrane	
		Top (nm)	Rear (nm)	Top (nm)	Rear (nm)
Oxalic acid	40	62	20	69	58
Oxalic acid	60	79	60	87	79
Oxalic acid–phosphoric acid	100	164	97	179	152
Phosphoric acid	185	282	357	343	355

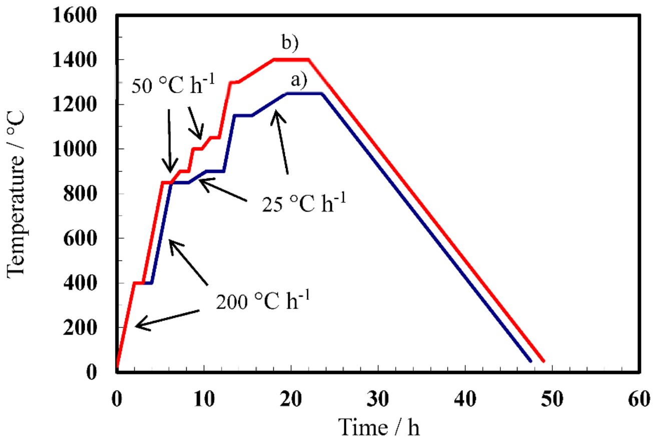

Figure S1. Elevated temperature process for crystallization of the alumina membranes formed at (**a**) 40 V and 60 V and (**b**) 100 V and 185 V.

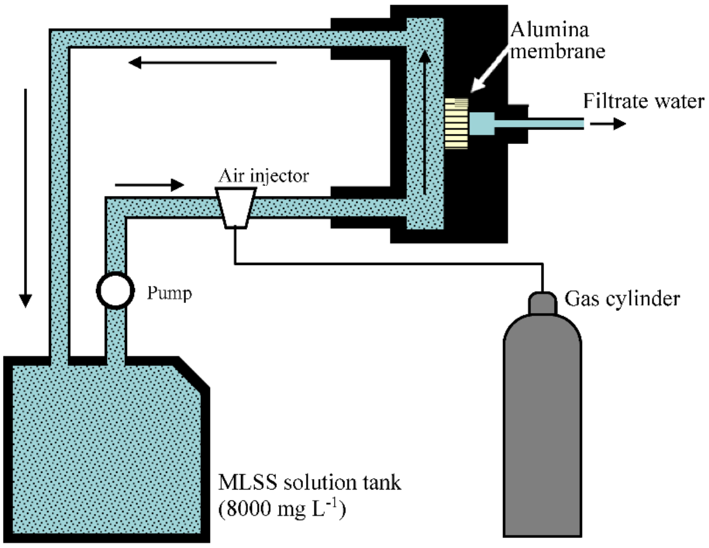

Figure S2. Setup for the filtration experiment.
